# Effects of idiopathic erythrocytosis on the left ventricular diastolic functions and the spectrum of genetic mutations: A case control study

**DOI:** 10.1097/MD.0000000000029881

**Published:** 2022-08-12

**Authors:** Alpay Yesilaltay, Hasan Degirmenci, Turker Bilgen, Duygu Yasar Sirin, Duygu Bayir, Pelin Degirmenci, Atakan Tekinalp, Seref Alpsoy, Yildiz Okuturlar, Burhan Turgut

**Affiliations:** a Division of Hematology, Department of Internal Medicine, Başkent University School of Medicine, İstanbul, Turkey; b Division of Hematology, Department of Internal Medicine, Acibadem Mehmet Ali Aydinlar University School of Medicine, İstanbul, Turkey; c Department of Cardiology, Tekirdag State Hospital, Tekirdag, Turkey; d Department of Nutrition and Dietetics, Tekirdag Namik Kemal University, School of Health, Tekirdag, Turkey; e Department of Molecular Biology and Genetics, Tekirdag Namik Kemal University, Faculty of Arts and Sciences, Tekirdag, Turkey; f Department of Internal Medicine, Tekirdag Namik Kemal University, Tekirdag, Turkey; g Division of Hematology, Department of Internal Medicine, Tekirdag Namik Kemal University, Tekirdag, Turkey; h Department of Cardiology, Tekirdag Namik Kemal University, Tekirdag, Turkey; i Department of Internal Medicine, Acibadem University School of Medicine, İstanbul, Turkey.

**Keywords:** cardiovascular diseases, mutation, polycythemia, ventricular function

## Abstract

**Background:**

We have aimed at exposing left ventricular diastolic functions and the presence of known genetic mutations for familial erythrocytosis, in patients who exhibit idiopathic erythrocytosis.

**Methods:**

Sixty-four patients with idiopathic erythrocytosis (mean age, 46.4 ± 2.7 years) and 30 age-matched healthy subjects were prospectively evaluated. The regions of interest of the erythropoietin receptor, hemoglobin beta-globin, von Hippel-Lindau, hypoxia-inducible factor 2 alpha, and Egl-9 family hypoxia-inducible factor genes were amplified by PCR. Left ventricular (LV) mass was measured by M-mode and 2-dimensional echocardiography. LV diastolic functions were assessed by conventional echocardiography and tissue Doppler imaging.

**Results:**

As a result of genetic analyses, genetic mutations for familial erythrocytosis were detected in 5 patients. It has been observed in our study that the risk of cardiovascular disorders is higher in patients. Interventricular septum thickness, left atrial diameter, and some diastolic function parameters such as deceleration time and isovolumetric relaxation time have been found to be significantly higher in idiopathic erythrocytosis group than in the controls.

**Conclusion:**

This study has shown that LV diastolic functions were impaired in patients with idiopathic erythrocytosis. In this patient group with increased risk of cardiovascular disorders, the frequent genetic mutations have been detected in 5 patients only. Therefore, further clinical investigations are needed as novel genetic mutations may be discovered in patients with idiopathic erythrocytosis because of cardiovascular risk.

## 1. Introduction

Erythrocytosis or polycythemia as commonly known refers to an increase in erythrocyte mass. Both acquired and familial erythrocytosis (FE), except relative erythrocytosis, are examined by subgrouping as primary and secondary.^[1]^ Primary-acquired erythrocytosis involves polycythemia vera (PV), which is characterized by the presence of JAK2 mutations, although these markers are not found in about 1% of patients with PV.^[2–4]^ Secondary-acquired erythrocytosis, however, occurs when erythropoietin (EPO) drives the production of red cells. This situation can develop in various cardiac, pulmonary, and renal diseases or from external hypoxic conditions.^[5]^ For familial polycythemias, a third subgroup defined as a mixed polycythemia has been identified other than its primary and secondary subgroups; this group includes erythrocytosis associated with the Chuvash polycythemia and other von Hippel-Lindau (VHL) gene mutations.^[6]^ Genes carrying mutations associated with familial polycythemias have been identified as erythropoietin receptor (EPOR), VHL, prolyl hydroxylase domain protein 2, and hypoxia-inducible factors 2 alpha (HIF2a) genes in the online Mendelian inheritance in man classification.^[7,8]^ Other polycythemias, defined by molecular lesions, are congenital methemoglobinemia, polycythemias caused by high oxygen and 2,3-bisphosphoglycerate deficiency.^[9,10]^ Once all the known causes of erythrocytosis have been ruled out, there remains a group of patients classified as having idiopathic erythrocytosis (IE). IE is characterized by an increase in red blood cell mass of unknown etiology.^[11]^ The frequency of IE is considered to be 1.1 for 1000 subjects. Diagnosing IE requires the exclusion of PV, secondary-acquired polycythemia, and various congenital primary and secondary polycythemias. It has also been reported that the risk of thrombosis is low in IE, and there may be spontaneous progress to acute leukemia or myelofibrosis.^[12]^

In patients with PV, it has been shown that blood rheology is associated with cardiovascular risk, and the mechanism of this is associated with impaired left ventricular (LV) function.^[13]^ Increased left ventricular mass (LVM) and deterioration in diastolic function parameters are risk factors for future heart failure development. In people with risk factors for the development of heart failure, asymptomatic LV dysfunction progresses to symptomatic heart failure over time.^[14]^ Approximately half of the cases of left heart failure are in the form of diastolic LV dysfunction.^[15,16]^ Heart failure that results from impairment of myocardial relaxation and compliance is called diastolic heart failure.^[17]^ LV diastolic dysfunction refers to increased cardiac filling pressures as a result of decreased LV relaxation and increased stiffness.^[18]^ It is important to monitor LV diastolic function parameters before diastolic heart failure develops. Measurement of transmitral blood flow with pulsed-wave Doppler and measurement of myocardial velocities from mitral annulus, septal, and lateral regions by tissue Doppler have become the preferred tool for noninvasively evaluating diastolic functions.^[19]^ Although there are studies investigating the relationship between LV diastolic functions in anemia and polycythemia vera, no similar studies have been found in IE.^[20,21]^

In our study, we have aimed at investigating and understanding whether isolated erythrocytosis would present as a risk factor for cardiovascular diseases. For this purpose, first, the primary- and secondary-acquired erythrocytoses were excluded, in which factors other than erythrocytosis could increase the risk of cardiovascular disease. Mutations in genes associated with FE have been investigated in all the cases. We have attempted to determine the presence of known mutations in proposed regions and tried to reveal new mutations related to erythrocytosis where possible. In addition, we have tried to determine the differences between the control group and the risk group with reference to the LV diastolic functions.

## 2. Materials and Methods

### 2.1. Patients

The study was conducted in the hematology and cardiology clinics of Tekirdag Namik Kemal University between January 2014 and January 2017. The inclusion criteria were as follows: isolated erythrocytosis (hemoglobin [Hb] > 18.5 g/dL in males or >16.5 g/dL in females), absence of JAK2 V617F and exon 12 mutations, and absence of any defined causes of acquired secondary erythrocytosis except smoking. Exclusion criteria were as follows: the patients under 18 years of age and patients with known coronary artery disease, cerebrovascular disease, or peripheral arterial disease. Furthermore, patients with chronic obstructive pulmonary disease, congestive heart failure, congenital heart disease, history of cardiac operation, atrial fibrillation, atrial flutter, and left bundle branch block were excluded from the study.

Sixty-four patients evaluated to have IE, and 30 healthy subjects that made up the control group were included in the study. Comprehensive clinical evaluations of the patients and controls who agreed to participate in the study were performed, and the patients and their families were questioned for erythrocytosis and related features. Moreover, routine laboratory findings (including blood count and lipid parameters) were recorded. Cardiovascular risk factors (diabetes, smoking, hypertension, and lipids) were designated/assessed in the patients and controls. EPO levels were measured in all patients. Written informed consent was obtained from all subjects. This study was approved by the Institutional Review Board of Tekirdağ Namik Kemal University.

### 2.2. Genetic analysis

**Figure 1.**
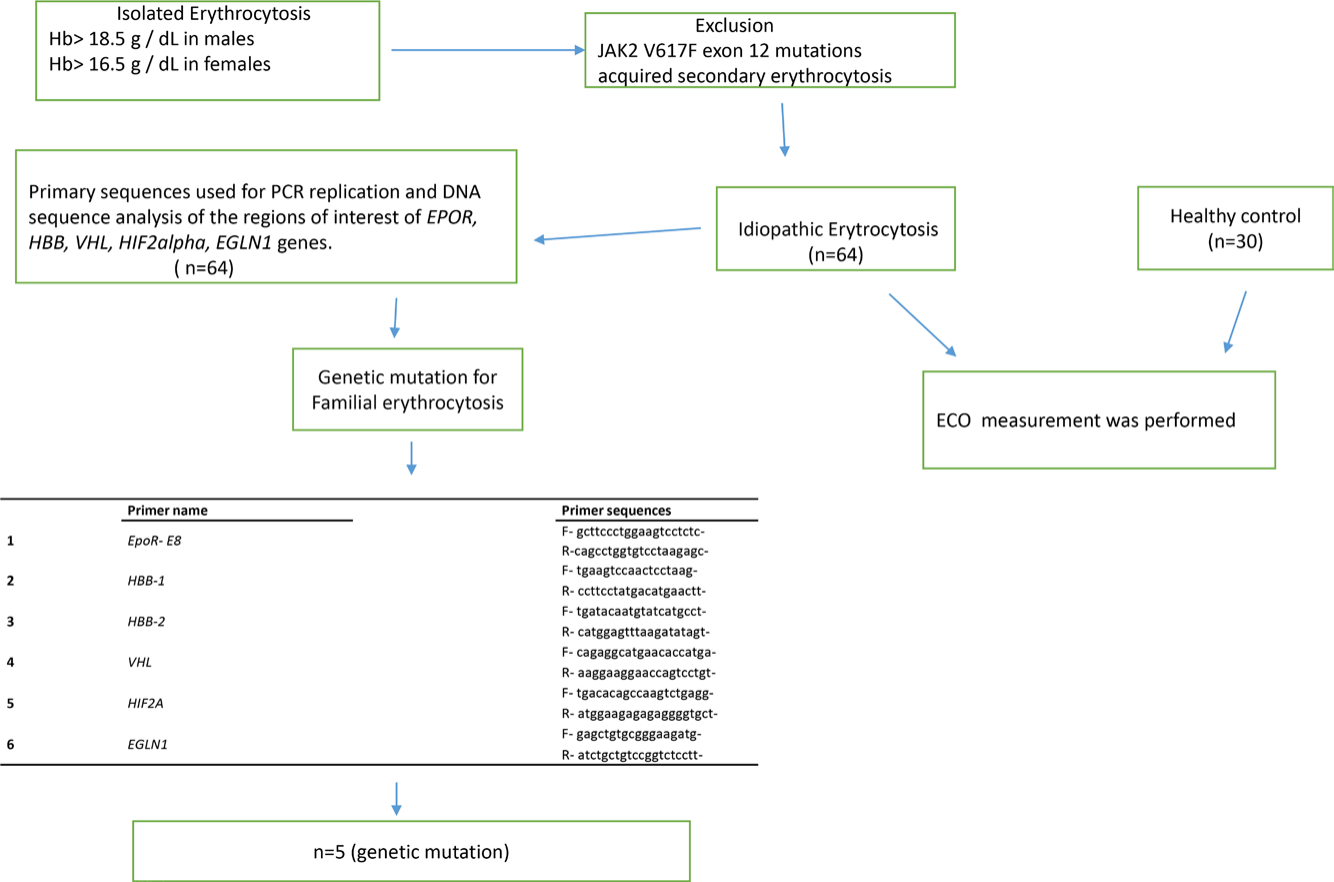
Mutation status of the patients in the isolated erythrocytosis except of JAK2 V617F exon 12 mutations and acquired secondary erythrocytosis. End of the analyses; 64 patients who are idiopathic erythrocytosis and 30 healthy control person were evaluated with echocardiography. DNA = XXX, ECO = XXX, EGLN1 = Egl-9 family hypoxia inducible factor, EPOR = erythropoietin receptor, Hb = hemoglobin HBB = hemoglobin beta globin, HIF2a = hypoxia-inducible factor 2 alpha, JAK2 = XXX, PCR = XXX, V617F = XXX, VHL = von Hippel-Lindau.

All of the cases included in the study group were assessed for FE-related genomic alterations by studying the mostly reported genes and mutations in the literature. Exon 8 of the EPOR gene (MIM number; 133171), entire coding and regulatory sequences of the hemoglobin beta-globin (HBB) gene (MIM number; 141900), the genomic region encompassing the Arg200Trp mutation that constitutes more than 90% of mutations of the VHL gene (MIM number; 608537), the genomic region including the most frequent mutations Ile533Val and Phe540Leu of the HIF2a gene (MIM number; 603349), and the genomic sequences containing the Cys127Ser and the Gln157His mutations in the first exon of the Egl-9 family hypoxia-inducible factor (EGLN1) gene (MIM number; 606425) were screened by direct sequencing. For this purpose, 5-cc peripheral blood samples were collected from the patients, and the samples were taken into K3-EDTA tubes, and genomic DNA isolations were carried out through a commercial kit (Roche, Germany) according to the manufacturer’s directives. The amount and purity of the isolated genomic DNA samples were determined. The regions of interest of the EPOR, HBB, VHL, HIF2a, and EGLN1 genes were amplified by PCR. The primer sequences used in PCR are given in Figure 1.

After the PCR, the products were checked by agarose gel electrophoresis. Then, PCR products were purified, and DNA sequence analysis reactions were performed. Repurified reaction products were subjected to capillary electrophoresis in an automated DNA sequencing device (GeXP, BeckmanCoulter) to determine the sequence of DNA fragments. Tekirdağ Namik Kemal University, the Scientific Technological Research and Application Center, performed the processes starting from DNA isolation to obtaining the sequence results. The results of the DNA sequence analyses were evaluated by comparing with the reference sequences, and genomic changes that could be related to FE were determined.

### 2.3. Echocardiography

Echocardiographic evaluation was performed using high-resolution B-mode ultrasound with a 2.5-MHz transducer by 2 experienced cardiologists who did not know the diagnosis of patients (GE Vivid S5: General ElectricVingMedSystems, Horten, Norway). While taking echocardiographic recordings, attention was paid to the appropriate room temperature and dim lighting. During the procedure, patients were asked to breathe normally. The interobserver variability for all measurements was found to be between 8.5% and 9.8%. Measurements of 20 patients were repeated 2 days later to evaluate intraobserver variability, and intraobserver variability for all measurements was between 6.8% and 7.8%.

### 2.4. Conventional echocardiography

Two-dimensional, M-mode, pulse-wave, and tissue Doppler echocardiography was performed for the measurement of left heart cavity dimensions, wall thicknesses, LVM, systolic, and diastolic function parameters. Left atrium diameters, LV systolic diameters, left ventricular diastolic diameters (LVDD), interventricular septum thicknesses (IVST), and posterior wall thicknesses (PWT) were measured according to the recommendations of the American Society of Echocardiography by 2-dimensional and M-mode echocardiography.^[22]^ The modified biplane Simpson method was used for LV ejection fraction measurements. The LVM was calculated with the Devereux formula^[23]^ as follows: 0.8 × {[(1.04 × [LVDD + IVST + PWT]^3^ − LVDD^3^)]} + 0.6.

### 2.5. Measurement of diastolic functions

In order to evaluate the conventional diastolic functions, diastolic transmitral flow was measured by pulsed-wave Doppler between the mitral leaflet tips at a level of 1 cm below the plane of the mitral annulus, in the apical 4-chamber view. The early (E) and late (A) phase peak flow velocities and their ratio (E/A), E wave deceleration time (DT), isovolumetric relaxation time (IVRT), isovolumetric contraction time (IVCT), and ejection time were measured.^[18]^

### 2.6. Tissue Doppler echocardiography

Tissue Doppler measurements were made using a 2.5-MHz variable frequency phased array transducer. To obtain LV tissue Doppler recordings, Doppler sample volume was placed in the septal and lateral corners of the mitral annulus from the apical 4-chamber view. The Nyquist limit was set to a velocity range from −20 to 20 cm/s. The monitor weep speed was set at 100 mm/s. Diastolic early (e), late (a), and systolic (s) waves were measured from septal and lateral regions of the mitral annulus, septal e/a, lateral e/a, E/septal e, and E/lateral e ratios.

### 2.7. Statistical evaluation

Statistical analysis of the data was carried out by using SPSS for Windows 18.0 software. The Shapiro-Wilk test was used to examine whether the data were normally distributed. All variables were found to be normally distributed. Continuous variables were expressed as mean ± standard deviation, and categorical variables as numbers and percentages. In statistical analysis, continuous variables between control and patient groups were compared with Student *t* test. Chi-square tests were used for comparison of categorical variables. Pearson correlation analysis was performed to investigate the relationship between Hb values and diastolic echocardiographic parameters. A *P* value less than .05 was accepted as genetic statistically significant.

## 3. Results

### 3.1. The results of the genetic analysis

**Figure 2.**
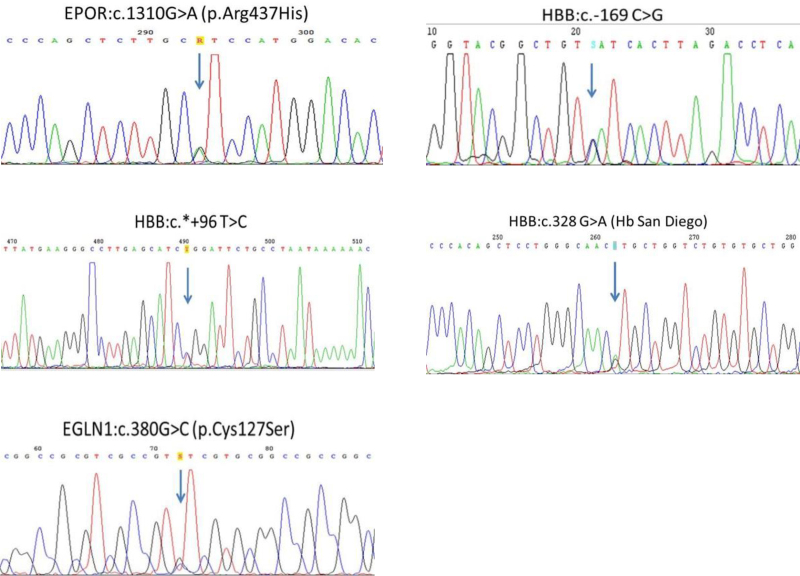
Genetic mutations for familial erythrocytosis in 5 patients. EGLN1 = Egl-9 family hypoxia inducible factor, EPOR = erythropoietin receptor, HBB = hemoglobin beta-globin.

As a result of genetic analyses, genetic mutations related to FE were detected in 5 patients (Fig. 1). EPO serum levels were heterogeneous among the patients; only 2 patients with Hgb San Diego had high EPO levels. We detected the EPOR gene 8 exon Arg437His change (EPOR: c.1310G>A [p. Arg 437His]) in heterozygous form in a male patient (Fig. 2). His highest hematocrit value was 53%. We have looked for the same genetic change in his relatives and detected the same change in 3 of his relatives. This change has been previously reported to be associated with erythrocytosis. A C>G change as heterozygous form in the promoter region of the beta-globin gene was detected in a male patient (Fig. 2). The patient was a 58-year-old man who had experienced syncope 2 years ago. His highest hematocrit value was 52%. This genetic change had not been reported in the medical literature previously and had not been present in any relevant databases. This genomic change was designated as HBB: c.-169 C>G according to the nomenclature reported by Human Genome Variation Society. A T>C change at the +96 3’UTR of the beta-globin gene HBB: c.+ + 96 T>C (rs34029390) was detected in heterozygous form in a male patient (Fig. 2). He was a 25-year-old patient who had been admitted with complaints of headache and had a hematocrit value of 52%. He did not have any history of arterial or venous thrombosis.

HBB: c.328 G>A (Hb San Diego) in heterozygous form was detected in a patient and his mother (Fig. 2). She was a 64-year-old woman who had under gone phlebotomy due to erythrocytosis for 20 years. A change in G>C at position 380 of EGLN1 gene (EGLN1: c.380G> C [p. Cys127Ser]) was detected in heterozygous form in a woman (Fig. 2). She was 45 years old, and her highest hematocrit value was 52%. Such a change had already been associated with erythrocytosis. There were no significant differences between the 2 groups in terms of age, gender, hypertension, diabetes, smoking, and lipid profile (*P* > .05 for all). Hb (18.1 ± 1.2 versus 14.5 ± 0.8 g/dL; *P* < .001) and hematocrit (58.2% ± 2.7% versus 43.9% ± 1.8%; *P* > .001) values were significantly higher in the IE group compared with the control group (Table 1).

**Table 1:** Age, gender, and cardiovascular risk factors of the patients and healthy subjects.

Variables	Patients (n = 64)	Controls (n = 30)	*P*
Age (mean ± SD)	46.33 ± 2.7	46.72 ± 3.1	.879
Gender (F/M), n (%)	3/61 (4.7/95.3)	1/29 (3.3/96.7)	.540
Hypertension, n (%)	26 (40.6)	11(36.7)	.320
DM, n (%)	6 (9.37)	2 (6.66)	.120
Smoking, n (%)	27 (42.2)	10 (33.3)	.680
TC (mg/dL)	193.0 ± 40.3	199.6 ± 47.4	.504
HDL-C (mg/dL)	42.1 ± 11.1	41.56 ± 10.2	.825
LDL-C (mg/dL)	118.4 ± 31.4	125.15 ± 40.8	.408
Triglyceride (mg/dL)	176.8 ± 88	176.3 ± 103.8	.981
Hemoglobin (g/dl)	18.1 ± 1.2	14.5 ± 0.8	**.001**
Hematocrit (%)	58.2 ± 2.7	43.9 ± 1.8	**.001**

### 3.2. Cardiovascular findings

There was no difference between the patients and control group members in terms of LV diameters, PWT, ejection fraction, mitral A wave, IVCT, ejection time, septal a, septal s, lateral a, lateral s, E/septal e, and E/lateral e and LVM (*P* > .05 for all). IVST (11.08 ± 0.87 vs 10.56 ± 1.13; *P* = .019), left atrial diameter (34.5 ± 2.8 vs 33.2 ± 2.6; *P* = .039), DT (128.84 ± 16 vs 121 ± 10.3; *P* = .019), IVRT (84.86 ± 20.46 vs 73.93 ± 6; *P* = .001), and LV myocardial performance index (MPI; 0.52 ± 0.14 vs 0.44 ± 0.03; *P* = .006) values were significantly higher in the patient group compared with the control group. Mitral E (62.80 ± 16.16 vs 74.48 ± 10.9; *P* = .001), E/A (0.93 ± 0.29 vs 1.14 ± 0.2; *P* = .001), septal e (8.75 ± 2.71 vs 10.52 ± 1.66; *P* = 0.001), septal e/a (0.89 ± 0.33 vs 1.1 ± 0.3; *P* = 0.006), lateral e (9.2 ± 1.7 vs 10.5 ± 1.3; *P* = 0.001), and lateral e/a (0.9 ± 0.3 vs 1.2 ± 0.26; *P* = 0.003) values were significantly lower in the patient group compared with the control group (Table 2).

**Table 2:** The findings of transthoracic echocardiography and tissue Doppler imaging in the patients and controls by comparison.

Variables	Patients (n = 64) (mean ± SD)	Controls (n = 30) (mean ± SD)	*P*
LVDD (mm)	46.58 ± 3.04	46.14 ± 2.17	.429
LVSD (mm)	32.25 ± 2.67	31.21 ± 1.78	.058
IVST (mm)	11.08 ± 0.87	10.56 ± 1.13	**.019**
PWT (mm)	10 ± 1.1	9.6 ± 0.9	.077
LAD (mm)	34.5 ± 2.8	33.2 ± 2.6	**.039**
EF (%)	64.2 ± 3.6	65 ± 2.1	.20
E wave (cm/s)	62.80 ± 16.16	74.48 ± 10.9	**.001**
A wave (cm/s)	69.15 ± 14.44	65.4 ± 9.3	.197
E/A ratio	0.93 ± 0.29	1.14 ± 0.2	**.001**
DT (ms)	128.84 ± 16	121 ± 10.3	**.019**
IVRT (ms)	84.86 ± 20.46	73.93 ± 6	**.001**
IVCT (ms)	51.64 ± 19.6	46.3 ± 5	.146
ET (ms)	264.6 ± 25.4	272.6 ± 14.2	.113
LV MPI	0.52 ± 0.14	0.44 ± 0.03	**.006**
Septal e (cm/s)	8.75 ± 2.71	10.52 ± 1.66	**.001**
Septal a (cm/s)	10.12 ± 2.26	9.8 ± 1.9	.568
Septal s (cm/s)	8.06 ± 1.94	7.45 ± 1.32	**.08**
Septal e/septal a	0.89 ± 0.33	1.1 ± 0.3	**.006**
Lateral e (cm/s)	9.2 ± 1.7	10.5 ± 1.3	**.001**
Lateral a (cm/s)	10.5 ± 2.8	9.6 ± 2.3	.125
Lateral s (cm/s)	9.00 ± 2.92	8.91 ± 2.17	.839
Lateral e/lateral a	0.9 ± 0.3	1.2 ± 0.26	**.003**
E/septal e	7.54 ± 1.95	7.24 ± 1.38	.464
E/lateral e	6.6 ± 1.9	7.12 ± 2	.257
LVM (g)	178 ± 29.4	166 ± 29	.071

While a positive correlation was found between Hb values and IVST (*r* = 0.245; *P* = .021), DT (*r* = 0.267; *P* = 0.012), there was a negative correlation between Hb values and E (*r* = −319; *P* = .002), E/A (*r* = −0.410; *P* = .001), septal e (*r* = −0.301; *P* = .004), septal e/a (*r* = −0.337; *P* = .001), and lateral e/a (*r* = −0.321; *P* = .002) (Table 3). Related parameters comparing 2 groups are shown in Table 2.

**Table 3:** Correlations of hemoglobin and diastolic function parameters.

Variables	Hemoglobin	
	*r*	*P*
IVST	**0.245**	**.021**
LAD	0.194	.068
E	**−0.319**	**.002**
E/A	**−0.410**	**.001**
DT	**0.267**	**.012**
IVRT	0.205	.054
Septal e	**−0.301**	**.004**
Septal e/septal a	**−0.337**	**.001**
Lateral e	−0.199	.061
Lateral e/lateral a	**−0.321**	**.002**
LV MPI	0.142	.183

## 4. Discussion

Our study was one of the very few genetic research studies conducted on familial erythrocytosis in our country. In addition to this fact, a rare cardiovascular risk assessment dimension was incorporated into the study. When one probes for similar research on a global scale, it becomes obvious that combined genetic and cardiovascular risk assessment studies have been limited both in number and in the number of patients included in such studies. In our study, we began by collecting data on mutations that have been known to have caused familial erythrocytosis. The second phase of our study consisted of how patients with IE presented themselves clinically. We assessed whether erythrocytosis caused any increase in cardiovascular risk. In connection with this, echocardiography results of patients both in the FE and in the control group were studied. Based on the echocardiography data available, it has been understood that cardiovascular risk increased in FE patients independent of the underlying genetic mutations.

In our study, 2 new genomic changes that had not been associated with FE and 3 known mutations associated with familial polycythemia have been detected. One of the 2 new genomic changes, HBB: c. + + 96 T> C (rs34029390) (HBB: c.*+96 T>C, rs34029390) was detected in heterozygous form in 1 individual. It has been reported that HBB: c. + + 96 T> C is a silent mutation that does not cause any change in the Hb level in neither heterozygous nor homozygous states, but its compound heterozygosity with beta-thalassemia mutations increases the severity of beta-thalassemia.^[24]^ The possible relationship of this mutation with erythrocytosis needs further investigation. In another case, a C>G change in heterozygous form in the promoter region of the beta-globin gene was detected. This change had not been previously reported in the medical literature and has not been existent in the relevant databases. Our medical literature search has revealed that HBB: c.-169 C>G change was involved in the binding site of the erythroid specific transcription factor, which has an effect on the expression of the beta-globin gene.^[25]^ Further studies are needed to show whether this mutation has an effect on the oxygen affinity of Hb.^[26]^

Except for those newly defined HBB mutations, Hb San Diego was also detected in 1 case and her son. Hb San Diego has been described in subjects of various origins, and only 1 case had been previously reported from Turkey so far.^[27]^ We detected EPOR exon 8 Arg437 His mutation (c.1310G>A) in 1 case and 3 family members. Pathological mutations in EPOR exon 8 lead to familial congenital polycythemia (PFCP). The diagnosis of PFCP has been made in a patient with IE in the presence of low or normal EPO serum level and a family history compatible with autosomal transition.^[28]^ The diagnosis of PFCP can be confirmed by detection of heterozygote EPOR mutation in only 12%–15% of cases. To date, mostly, mutations of EPOR, which cause PFCP, within exon 8 have been described.^[29]^ Until now, 2 cases with c.1310G>A variant have been described in the literature. One of them was a male 24 years of age who required regular phlebotomy. The other one was a 52 years old patient with a clinical history of recurrent venous thrombosis with normal Hb and hematocrit levels and no familial history of hematologic disorder.^[30]^ Our case represents the third subject in literature detected with this variant.

We detected EGLN1:c.380G>C (p.Cys127Ser) missense mutation in 1 case, which had been first detected by Lorenzo et al.^[31]^ The EGLN1 mutations cause secondary FE of the familial erythrocytosis type 3 online Mendelian inheritance in man classification. Already, EGLN1 encodes PHD2, which is the principal negative regulator of (HIFs) and EPAS1 encoding HIF-2a.^[32]^ It has been reported that EGLN1 c.380G>C together with EGLN1 c.12C>G as a haplotype can be a component of a complex array of genetic adaptations involving both PHD2 and HIF-2a that results in lower Hb in Tibetan highlanders.^[33]^

In PV and other Ph-negative myeloproliferative neoplasms, there is a high incidence of thrombosis due to an acquired thrombophilic state.^[34]^ The mechanism of the acquired thrombophilic state in these diseases is multifaceted. Prothrombotic features of myeloproliferative neoplasm clone–derived blood cells, not only erythrocytes but also platelets and leukocytes and procoagulant changes of normal vascular cells, in response to inflammatory stimuli, can be considered as mechanisms of this thrombophilic state. In fact, hyperviscosity due to erythrocytosis does not seem as an important factor for thrombosis in PV.^[35]^ Chuvash polycythemia causes thrombotic and hemorrhagic vascular complications that lead to early mortality usually before the age of 40 years.^[36–38]^ Apart from Chuvash polycythemia, the association of FE and IE with thrombosis and cardiovascular complications are not clear.^[39]^

In previous publications, it had been demonstrated that PV and some types of anemia had impaired LV diastolic functions.^[21]^ Aging, coronary artery disease, diabetes, hypertension, and atrial fibrillation are important factors contributing to diastolic dysfunction.^[40]^ To clearly assess the contribution of erythrocytosis in diastolic dysfunction, we have excluded individuals with coronary artery disease and atrial fibrillation. Also, there was no difference between the groups in terms of age, gender, hypertension, diabetes, and smoking habits. In this study, we found some parameters such as decreased mitral E wave, transmitral E/A ratio, septal e, septal e/a, lateral e, and lateral e/a ratio and increased DT and IVRT values, as evidences of decreased LV relaxation. In the presence of mild diastolic dysfunction, relaxation of the left ventricle is impaired; early filling of the left ventricle in diastole becomes difficult; accordingly, mitral E wave, and septal e and lateral e wave amplitudes decrease, and E wave DT and IVRT get prolonged. These findings show that stage 1 diastolic dysfunction exists in patients with IE. In addition, the atrial contribution increases exaggeratedly due to late filling of the left ventricle in diastole, thereby increasing the mitral A wave amplitude. As a result, mitral E/A, septal e/a, and lateral e/a ratios decrease.^[41]^

We have not found any difference between the cases with erythrocytosis and healthy controls for cardiovascular risk factors such as hypertension, lipid parameters, diabetes, and smoking. Furthermore, we assessed cardiovascular status of all subjects with transthoracic echocardiography. It has been suggested that transthoracic echocardiography can be used a detection method for LV hypertrophy, which is considered as a cardiovascular risk factor in asymptomatic adults who have hypertension.^[42]^ LV hypertrophy is an important risk factor for diastolic dysfunction. Therefore, we have performed M-mode and Doppler echocardiography for our patients and healthy controls. M-mode echocardiography showed a higher thickness of interventricular septum in our cases than the control subjects. This was thought to have been caused by increased workload of heart due to increased viscosity in the patient group. In the study of Kayrak et al.,^[21]^ LVM had been found to have increased in patients with polycythemia vera. Although the septum thicknesses increased, the LVM data of patients were not different from those in the control group in our study. We think this is due to the fact that viscosity is lower in patients with IE compared with polycythemia vera and that the LV workload is less.

Mitral E/A ratio has been found to be lower in the patients with pulsed-wave Doppler echocardiography. Additionally, a prolongation of IVRT and IVCT and the low value of mitral annulus septal e, in tissue Doppler echocardiography, have been detected, which were thought to be associated with slowing of the blood flow rate due to increased hematocrit levels. As a result, increased cardiac workload may lead to deterioration of myocardial relaxation over time, resulting in diastolic dysfunction.

Increased MPI is an indicator, showing that both LV diastolic and systolic functions are impaired together. MPI has been shown to have increased in PV.^[21]^ In our study, we have found an increase in MPI in the IE patients. This suggests that the LV systolic and diastolic functions begin to deteriorate together in these patients due to increased viscosity.

In conclusion, mutations that are known to cause familial erythrocytosis have been shown, and beta-globulin gene changes that are likely to cause secondary familial erythrocytosis have been identified in our study. In addition, it has been determined that echocardiographic findings related to cardiovascular workload and cardiovascular morbidity and mortality were present prevalently in IE cases.

Even though FE patients are diagnosed rather prevalently in internal medicine/hematology clinics, no underlying pathogenetic cause is studied due to lack of established algorithms. FE is only differentially diagnosed from PV, with no follow-up procedures with reference to genetic aspects and cardiovascular risks involved. This means that such a relatively prevalently seen and important disease is being overlooked at the expense of patients. FE patients are under serious cardiovascular risks apparently with the AD genetic transfer perpetuating the risks for the next generations and the society, unless awareness is augmented and due care is given to such patients.

## Acknowledgments

We thank Orhan Cem Deniz and Yasemin Gundogdu who provided medical writing supports.
